# An Experimental Research into the Nature of Nitrous Oxid and of Ether Anesthesia*Read before the American Association of Anesthetists, Detroit, 1916.

**Published:** 1917-08

**Authors:** George W. Crile

**Affiliations:** Cleveland, Ohio


### AN EXPERIMENTAL RESEARCH INTO THE NATURE OF NITROUS OXID AND OF ETHER ANESTHESIA *

BY GEORGE W. CRILE, M.D., CLEVELAND, OHIO

If we view man and the lower animals as adaptive mechanisms, governed by the universal laws of physics and chemistry, then we must believe that every phase of human or animal behavior is occasioned by and in turn occasions physical and chemical changes in the body. Therefore an understanding of the vast alterations in the behavior of the anesthetized organism—changes which may range from the slightest depression of mental power through progressively deeper stages of unconsciousness to death, necessitates a search for the physical and chemical changes caused by the anesthetic. Accordingly, in this study we shall consider (*a*) the gross behavior of the anesthetized individual in a consideration of the relation of anesthesia to normal sleep; (*b*) the influence of ether and of nitrous oxid on the histologic structure of certain organs; (*c*) the relation of ether and of nitrous oxid to the hydrogen-ion concentration of the blood and to reserve alkalinity; (*d*) the relation of ether and of nitrous oxid to the infections, and finally (*e*) the clinical bearing of these studies.

(*a*) *The Relation of Inhalation Anesthesia to Normal Sleep.* The outward similarity in the appearance of sleep and of anesthesia needs no comment. The manner in which a child becomes unconscious under a sufficiently concentrated flow of nitrous oxid over its face closely resembles the manner of which that healthy normal child falls asleep. That sleep is merely the depression of consciousness and that sleep only partially suspends the function of the brain is indicated by slight responses to stimuli, such as the shifting of posture, the moving when called or when bright

* Read before the American Association of Anesthetists, Detroit, 1916.

light strikes the eye, the response to tickling, etc. In other words, the receptors are still active during sleep. The activity of the receptors in anesthesia is evidenced as clearly by the increased pulse and respiration in response to trauma; and, if the anesthesia be light, by increased muscular tone, even by muscular movements. Another similarity is found in the resemblance between dreams during nitrous oxid anesthesia and the dreams of normal sleep.

(*b*) *The Influence of Ether and of Nitrous Oxid on the Histologic Structure of Various Organs.* As far as nitrous oxid is concerned, here also is found another resemblance to normal sleep. During normal sleep the histologic changes in the brain, the suprarenals and the liver caused by exertion, emotion, infection, acid injection, etc., are repaired; during nitrous oxid anesthesia also, the lesions in the brain cells due to these causes are repaired, and, as will be seen later, nitrous oxid exerts a measurably protective action on the suprarenals and liver as well. This protective action of nitrous oxid was strikingly demonstrated in a series of experiments on insomnia. A group of rabbits were kept awake continuously for approximately one hundred hours. At the end of this period, their brain cells showed the typical physical changes of exhaustion. In another group these changes were partially retored after a single seance of sleep; while in still another group, in which the rabbits were allowed no normal sleep, but were placed under nitrous oxid anesthesia for one hour out of every six during a period of approximately one hundred hours, the brain cells, as in the rabbits allowed to sleep, showed marked restoration, some cells, indeed, being in a *plus normal state.*

A comparative study of the effects of ether and of nitrous oxid was made by keeping dogs under continuous anesthesia for four hours. After four hours' continuous ether anesthesia, striking histologic changes were found in the brain, the suprarenals and the liver; while after four hours' continuous nitrous oxid anesthesia, there were practically no changes in the brain, and but slight changes in the liver and suprarenals.

(*c*) *The Relation of Ether and of Nitrous Oxid to the Hydrogen-ion Concentration of the Blood and to Reserve Alkalinity.* In a series of H-ion concentration tests made for me by Dr. M. L. Menten, it was found that the H-ion concentration of the blood was increased by ether and by nitrous oxid, and that the acidity increased more rapidly under nitrous oxid than under ether; but that the total acidity in either case was proportional to the depth of anesthesia.

In measurements of the reserve alkalinity of the blood made for me by Dr. W. B. Rogers, he found that during anesthesia the reserve alkalinity gradually decreased, the total decrease being greater under ether than under nitrous oxid. He found also that the alkalinity of the spinal fluid and of the bile was altered during anesthesia.

It is significant to note here the fact that the histologic lesions in the brain, the suprarenals and the liver, caused by ether and by nitrous oxid, though varying in degree, are identical in kind with those caused by the intravenous injection of acids, as with those resulting from insomnia, exertion, emotion, infection or physical injury.

We must conclude, therefore, that the histologic lesions of inhalation anesthesia are acid lesions; and since acid lesions are restored only during sleep, that the same must be true of the lesions of anesthesia.

As long as fifteen years ago it was believed that all anesthetics interfered with the use of oxygen, this being the method by which anesthesia was produced. It is true that deep asphyxia may cause anesthesia, but in such asphyxia as is due to obstruction of the larynx, the patient remains conscious and feels pain, though he is markedly cyanotic, while under good inhalation anesthesia, the patient is pink and unconscious. It would seem, therefore, that interference with oxidation alone is an insufficient explanation of anesthesia. On the other hand, the postulate that anesthesia is an induced acidity is not only supported by the laboratory studies we have mentioned, but by the clinical phenomena of anesthesia as well.

It may be in place to refer here to Lillie's notable studies of the mechanism of ether anesthesia. He has demonstrated that the addition of ether to the seawater in which arenicola are living causes changes in the semipermeable membranes, as a result of which these membranes become less permeable to the passage of ions. As life itself is dependent on the permeability of the cells of the organism to the passage of ions, this action of ether suggests a further explanation of anesthesia, though its application has not been worked out in detail.

(*d*) *The Relation of Ether and of Nitrous Oxid to the Infections.* We have already referred to the protective action of nitrous oxid in the presence of the stimuli of continued consciousness as in prolonged insomnia; and other histologic studies have shown that morphin also protects the brain, suprarenals and liver from the destructive action of prolonged or intense stimuli. It follows, then, that since the effects of an infection on the brain, suprarenals and liver are identical with the effects of insomnia, emotion, etc., then the administration of nitrous oxid and morphin will in part protect the organism against the damaging effect of infection.

*Summary of Experimental Findings.* Nitrous oxid and ether anesthesia alike cause an increase in the H-ion concentration of the blood, the spinal fluid and the bile; and progressively decrease the reserve alkalinity of the blood. Ether causes marked histologic changes in the brain, suprarenals and liver, these changes being identical in kind with the histologic lesions caused by acid injection, or activation of any kind. Nitrous oxid and morphin each measurably protect the brain against histologic changes due to infection, while ether increases the damaging effects of infection.

(*e*) *The Clinical Bearing of These Studies.* The identity of the phenomena of anesthesia with the succession of clinical symptoms which accompanies an increasing acidosis, combined with the fact that the histologic changes produced by nitrous oxid and by ether are identical with those caused by acidosis, supports the postulate that anesthesia is an induced acidity. If the acidity is slight, anesthesia is light and the patient responds to even slight stimulation. As the acidity increases, the anesthesia deepens—first, associative memory is lost, but the cutting of the skin still causes involuntary movements; then muscular tone is lost, and even the strong contact stimuli of a surgical operation can not drive their impulses through the brain to the muscles; and finally, the decreasing alkalinity may so nearly approach the neutral point that even the circulatory and respiratory centers, which are especially adapted to respond to the stimulus of increased H-ion concentration, fail to respond; respiration and circulation are suspended and acid death—anesthetic death—follows.

These studies explain why a patient, whose reserve alkalinity has been seriously reduced by exertion, emotion, physical injury or infection, or, by reason of starvation, interference with respiration or impairment of the liver or kidneys, is approaching acidosis, does not take an inhalation anesthetic well; why there is much nausea and slow recovery from anesthesia in some cases and death in others, and why, in particular, children who are near acidosis always pass into that state and die unexpectedly.

These facts show how necessary it is for the surgeon and the anesthetist alike to realize that during the operation each is draining the store of reserve alkalinity. If the surgeon employs a local anesthetic, uses gentle manipulation and produces the least possible trauma, he conserves his patient and demands from the anesthetist less of the damaging inhalation anesthetic. The anesthetist in turn conserves the patient by using the lightest possible even anesthesia administered with the least psychic trauma.

These studies show that for the bad risk patient, nitrous oxid anesthesia is to be preferred to ether, and that analgesia with local anesthesia should be employed, with general anesthesia only when it is demanded by certain phases of the operation.

These researches suggest the value of a mechanistic view of the phenomena of anesthesia and attach a high importance to the work of the anesthetist.

In my own clinic we have now administered nitrous oxid anesthesia in over 15,000 cases without a death; and moreover, as our knowledge and experience accumulate, we are able with increasing accuracy to adapt the anesthetic to the individual.—*The Dental Summary.*

